# Predictors of health care drop-out in an inception cohort of patients with early onset rheumatoid arthritis

**DOI:** 10.1186/s12891-017-1670-6

**Published:** 2017-07-28

**Authors:** Irazú Contreras-Yáñez, Virginia Pascual-Ramos

**Affiliations:** Department of Immunology and Rheumatology, Instituto Nacional de Ciencias Médicas y Nutrición Salvador Zubirán, Vasco de Quiroga 15, Colonia Belisario Domínguez, Sección XVI, Tlalpan, 14500 México, DF Mexico

**Keywords:** Quality of health care, Rheumatoid arthritis, Patient attitude to health

## Abstract

**Background:**

RA patients who eventually dropped out of treatment and out of the health care system had potentially disastrous consequences for their health-related quality-of-life outcomes. Objectives of the study were to identify predictors of health care drop out (HDO) in an inception and ongoing cohort of patients with recent onset RA.

**Methods:**

Charts from patients attending an early arthritis clinic from February 2004 to December 2015, and standardized follow-up evaluations were reviewed. Patients with HDO (cases) were defined when they did not return back to the clinic for a schedule visit for at least one year. Persistence with therapy was defined as length of time patients complied with RA-treatment. A case-control nested within a cohort design was used to compare baseline and cumulative (up to HDO or equivalent follow-up) variables between cases and paired controls (patients compliant with scheduled visits). Cox regression analysis was used to investigate predictors of HDO. The study was approved by the Institutional Review Board and patients gave written informed consent to have their data published.

**Results:**

Data from 170 patients (89.4% female, [mean±SD] age: 38.2±12.6 years) with ≥1 year of follow-up were analyzed; up to December 2015, (median, interquartile rage) follow-up was 86.6 months (43.2–123) during which 35 (20.6%) patients had HDO after 41.1 months (12.1–58.7). Baseline and cumulative variables related to disease activity, treatment and persistence with therapy entered regression models; cumulative number of flares, number of disease-modifying anti-rheumatic drugs /patient and persistence <50% emerged as predictors of HDO. Five cases returned back after (median, range) drop out time of 3.8 years (2.3–5.8); they exhibited higher disability and poorer function than paired controls and outcomes were sustained up to their last follow-up.

**Conclusions:**

In a real clinical setting of an EAC, failure to control disease activity, intensive treatment and poor persistence with therapy predicted HDO. Abandonment of health care had a negative impact on patient outcomes and was sustained even after health care was reinitiated.

## Background

Rheumatoid arthritis (RA) is a chronic inflammatory disease that frequently results in disability and morbidity [1–3]. Patients from Latin-America present distinctive epidemiological, serological and clinical disease features. The literature reports a lower prevalence [4], a younger age at presentation [4, 5] and a less severe clinical expression [5] in this population compared with Caucasians. Nonetheless, these patients are frequently uninsured, have low socioeconomic status and are less educated than RA patients from developed countries [4]. All of these variables are known to impact patient commitment to physician consultations and their prescribed treatment(s).

In 2004, we established an early arthritis clinic (EAC) in a referral center for rheumatic diseases in México City, México, with the aim of identifying patients with recent-onset RA. Once enrolled in the inception cohort, patients underwent evaluations scheduled at regular intervals and were prescribed treatment, according to a ‘treat to target’ strategy. In addition, their compliance with treatment was prospectively assessed. Similar to previous published observations [6–16], poor compliance with therapy was frequent and was associated with worse physician- and patient-reported outcomes [5, 17, 18]. Moreover, when patients concomitantly identified a lack of financial resources and a lack of available medications at the drug store as reasons for nonadherence to therapy, they were at risk for recurrent abandonment of therapy and experienced even more unfavourable outcomes [19].

Ultimately, the impact of inadequate therapy behaviour in RA patient outcomes may be further amplified by the fact that almost all individuals with poor drug compliance eventually dropped out of treatment and out of the health care system completely [20], which had potentially disastrous consequences for their health-related quality-of-life outcomes.

The study objectives were to: Identify and describe patients from an inception ongoing cohort with recent onset RA at inclusion, who dropped out of health care during their follow-up (objective 1); Compare their baseline characteristics and cumulative disease activity, treatment and persistence with therapy with those from matched patients currently attending the clinic (objective 2); Identify predictors of health care drop out (HDO) (objective 3), and investigate the impact of HDO on outcomes (objective 4).

## Methods

### Study population

The *Instituto Nacional de Ciencias Médicas y Nutrición Salvador Zubirán* belongs to the National Institutes of Health of México. Patients enrolled in the EAC had a disease duration of <1 year when first evaluated, and no specific rheumatic diagnosis except for RA. Patients were evaluated by a single rheumatologist every two months during the first two years of follow-up, and every two, four or six months (fixed for all patients from the baseline evaluation) thereafter. Patients had partial health coverage and paid for their physician’s consultations, laboratory investigations and for their treatment that was prescribed by the rheumatologist in charge of the clinic, and was “treat to target” oriented; accordingly, traditional disease-modifying anti-rheumatic drugs (DMARDs) were used in 98% of the patients with/without corticosteroids (50% of the patients received low doses of oral corticosteroids during their follow-up). To December 2015, the cohort comprised 180 RA patients with variable follow-up recruited from February 2004 onward; 172 had at least one year of follow-up.

### Standard rheumatic evaluations

At study enrollment, complete medical history and demographic data were recorded, in addition to the type(s) of disease-specific autoantibodies; also, American College of Rheumatology (ACR) 1987 classification criteria for RA were applied. Medical evaluations were standardized and included at least 66 swollen and 68 tender joint counts, acute reactant-phase determinations (erythrocyte sedimentation rate [ESR] and C-reactive protein [CRP]), patient- and physician-reported outcomes, comorbidity established by record review, and treatment assessments (name[s], dose[s] and schedule[s] of all drug[s] they were taking since last visit), along with an evaluation of persistence, at six-month intervals (fixed for all patients). Accordingly, a minimum of six months of follow-up data were required to evaluate persistence with therapy. At follow up evaluations, the Disease Activity Score in 28 joints was calculated using ESR (DAS28-ESR) [21].

### Definitions

*Cases* (HDO patients) were defined as individuals who did not return to the EAC for a scheduled visit for at least one year. When patients failed to attend a scheduled follow-up, a visit was rescheduled within 2 weeks. In addition, attempts to re-schedule missing evaluations were done at regular intervals unless the patient manifested wiliness to abandon health care.

*Controls* were defined as individuals who were compliant with their scheduled visits. Controls also included patients lost to follow-up but who returned to the clinic within one year since their last visit.

*Non-persistent patients* were identified through an interview from 2004 to 2008 and defined as omission of at least one disease-modifying anti-rheumatic drug (DMARD) and/or corticosteroids for at least seven consecutive days. From 2008 to date, non-persistence was defined according to the Concordance Questionnaire (CQ), if in item 10 “In the past 6 months how often you did completely stop taking your DMARDs?” box 2 (Sometimes), box 3 (Almost always) or box 4 (Always) were marked [18, 19, 22]. Treatment modifications due to adverse events and/or indicated by a different physician for any reason (insufficient response, pregnancy, scheduled surgery, etc.…) were not considered under the non-persistence definition.

*Persistence* was defined (within each patient) as the percentage of the patient entire follow-up, he/she was persistent with therapy. In addition, persistence <50% was defined when patient was persistent with therapy less than 50% of his/her entire follow-up; this variable was created based on previous publications that have demonstrated an association between (better) persistence and more favorable outcomes (19, 22).

*Disease activity* was evaluated as per DAS28-ESR. Briefly it is a complex index, validated to objectively assess disease activity and patient’s response to treatment; it is based on a count of 28 specific swollen and tender joints, the ESR (in mm/H) and a general health visual analogue scale filled by the patient (0 to 100 mm). DAS28-ESR is expressed into one continuous measure with a score ranging from 0 to 9.4. In addition, specific cut-offs have been established to classify four disease activity statuses: remission, low disease activity, moderate and high disease activity [21].

*Patients in remission* were defined as individuals who had a DAS28-ESR at <2.6 [21]; sustained remission was defined if the patient’s DAS28-ESR was maintained at <2.6 for at least one year of follow-up.

*Flare* was defined at a DAS28 ≥ 2.6, scored in at least one evaluation after the patient had achieved remission.

### Statistical analysis

Descriptive statistics, Student’s *t* and Χ^2^ tests were used, as appropriate. Sociodemographic data are presented as (mean ± SD), while disease and treatment characteristics are described as median and interquartile range (Q_25_-Q_75_).

To achieve objective 2 (compare baseline characteristics and cumulative disease activity, treatment and persistence from patients with HDO and matched patients currently attending the clinic), baseline characteristics were first compared among the 35 cases (patients with HDO) identified and their respective 135 controls. In addition, a case-control study nested in the cohort was designed to compare cumulative disease activity, treatment and persistence between both groups. Each HDO patient was paired with two controls according to age (±5 years), sex, rheumatoid factor (RF) and antibodies to citrullinated peptides (ACCP), and follow-up to HDO (or equivalent follow-up in controls). Cumulative disease activity, treatment and persistence were compared between groups; the analysis was repeated with the same variables computed to the year before HDO.

To investigate the impact of HDO on outcomes, the health assessment questionnaire (HAQ), Short-form 36 Health Survey (SF-36) and presence of erosions from five patients who met the criteria for HDO but who returned to the EAC were compared with those from 15 controls (i.e., 3 to 1) adherent to visits. They were paired according to age (±5 years), sex, RF and ACCP, baseline erosions, cumulative disease activity and treatment up to HDO (or equivalent). Outcomes were evaluated when patients returned to the clinic (or equivalent follow-up) and at the last follow-up.

Cox regression analysis was used to investigate predictors of HDO (objective 3); the authors were particularly interested in variables related to cumulative disease activity and patient therapy behaviour (independent variables) as potential contributors to HDO, in addition to baseline variables. Variables included in the different models tested were selected based on their statistical significance in the univariate analysis (*p* ≤ 0.20) and on their clinical relevance. Age was forced into the models as it has been found to be a predictor of poor adherence and, consequently, worse outcomes [22]. Correlations between variables to be included (as disease activity and treatment) were also examined and the final number of variables (up to five) was limited by the number of outcomes of interest (35 patients with HDO) to avoid overfitting the models. Significant variables were retained using backward stepwise (conditional) selection.

All statistical tests were two-sided and evaluated at the 0.05 significance level. Statistical analysis was performed using SPSS version 17.0 (IBM Corporation, USA).

### Ethics approval

The present study was approved by the Institution’s internal review board. Written informed consent was obtained from all the patients entering the cohort.

## Results

### Characteristics of the study population and comparison of baseline characteristics between cases and controls (objectives 1 and 2)

To December 2015, charts from 172 patients with at least one year of follow-up were reviewed by one single data extractor: 35 were defined as cases and 135 as controls (two died: one from pneumonia and the other from an unknown cause; their data were included in the analysis as part of the controls). Two additional patients dropped out from the EAC early after their inclusion because they were unable to commit to scheduled visits during the first two years and their data were excluded. The final number of patients for which data were analyzed was 170.

As summarized in Table 1, patients were primarily middle-age female, with short disease duration, high disease activity and substantial disability at cohort inclusion. The majority of them (97%) met ≥4 ACR classification criteria for RA. They frequently had disease-specific autoantibodies and, at referral, almost one-half (48.8%) were receiving DMARDs and had at least one comorbid condition (48.2%); 32.4% of the patients were taking low doses of oral corticosteroids.

**Table 1:** Baseline population characteristics and comparison between cases and controls

	RA population, *N* = 170	HDO patients, *N* = 35	Patients with continuous follow-up, *N* = 135	*p*-value
Socio demographics				
Female gender, N° (%) of patients	152 (89.4)	32 (91.4)	120 (88.9)	1
Age at baseline, years	38.2 ± 12.6	37.5 ± 9.7	38.4 ± 13.2	0.6
Years of formal education	11 ± 3.8	10.1 ± 3.4	11.3 ± 3.8	0.10
Disease characteristics*				
Disease duration, months	5.3 (3.5–7)	4.6 (3–6)	5.5 (3.5–7.2)	0.10
% of patients with RF /ACCP	82.9/84.7	77.1/77.1	84.4/86.7	0.3/0.2
DAS28	6 (4.9–7)	6.2 (5.3–7.6)	5.9 (4.7–6.8)	0.06
N° swollen joints (0–28)	13 (8–18)	15 (10–21)	13 (8–17)	0.05
HAQ	1.4 (0.9–2.1)	1.5 (1–2.1)	1.4 (0.7–2.1)	0.3
SF-36	36.4 (26.2–53)	31.3 (21.9–45.2)	38.8 (27.6–54.9)	0.02
N° (%) patients with comorbidities	82 (48.2)	11 (31.4)	71 (52.6)	0.04
Charlson score*	1 (1–1)	1 (1–1)	1 (1–1)	0.09
Referral treatment				
N° (%) of patients on corticosteroids	55 (32.4)	9 (25.7)	46 (34.1)	0.4
N° (%) of patients on DMARD	83 (48.8)	14 (40)	69 (51.1)	0.3
N° of DMARD/patient^*,a^	1 (1–2)	1 (1–1.2)	1 (1–2)	0.2

Data presented as (mean ± SD) unless otherwise indicated

*Data presented as median (Q_25_-Q_75_)

^a^Restricted to patients on DMARDs

To December 2015, the median length of follow-up in the cohort was 86.6 months (43.2 to 123 months), during which 35 (20.6%) patients dropped out of health care after 41.1 months (12.1 to 58.7 months); 28 (80%) patients dropped out of health care during the first five years.

Comparison of baseline characteristics between cases and controls are presented in Table 1. Patients from the former group had a greater number of swollen joints, lower SF-36 scores (physical and emotional components) and had fewer comorbidities than their counterparts. Additionally, cases tended to have lower educational levels and shorter disease duration than their counterparts.

### Comparison of cumulative disease activity, treatment and persistence between cases and paired controls (objective 2)

Data from 35 cases paired with 70 controls are summarized in Table 2. The variables compared included cumulative data up to HDO or equivalent follow-up among the controls. Up to HDO, patients from the former group showed higher cumulative disease activity, achieved sustained remission less frequently and experienced a greater number of disease flares/patient than their counterparts. They also tended to score lower on the SF-36. There were no differences between the groups regarding neither the number of visits/patient nor in the number of patients taking corticosteroids, although the number of DMARDs/patient tended to be higher in patients from the former group. Interestingly, patients with HDO had shorter persistence (defined as the percentage of their entire follow-up persistent with therapy) and tended to be less frequently ‘always persistent’ than their counterparts. Finally, in this subpopulation of 105 patients, baseline sociodemographic variables, disease characteristics and comorbidities were compared between cases and controls: lower SF-36 scores were confirmed in cases versus controls (median 31 [22 to 45] versus 39 [27 to 60]; *p* = 0.02) and a tendency toward lower levels of education ([mean ± SD] years of scholarship: 10.1 ± 3.4 versus 11.5 ± 3.8; *p* = 0.07), higher DAS28-ESR (median 6.2 [5.3 to 7.6] versus 5.9 [4.5 to 6.8]; *p* = 0.06) and to a higher number of swollen joints (median 15 [10 to 21] versus 13 [8 to 17]; *p* = 0.06).

**Table 2:** Cumulative disease activity, quality of life, treatment and persistence with therapy between cases and controls

	Patients with HDO, *N* = 35	Patients with continuous follow-up, *N* = 70	*p*-value
Cumulative disease activity and function			
DAS28	3.1 (2.3–4.2)	2.4 (1.9–3.2)	0.02
N° (%) of patients with sustained remission at outcome	18 (51.4)	57 (81.4)	0.03
N° of disease flares/patient^a^	1 (1–2)	0 (0–1)	0.000
SF36 score	74 (63–82)	81 (70–89)	0.06
N° of schedule visits	7 (3–9)	7 (3–9)	0.94
Cumulative treatment			
N° (%) of patients on corticosteroids	13 (37.1%)	30 (42.6)	0.68
N° of DMARD/patient	2 (1–2)	1 (1–2)	0.06
Cumulative patient’s treatment behaviour^b^	*N* = 30	*N* = 60	
N° (%) of patients always persistent^b^	8 (26.6)	26 (43.3)	0.17
% of follow-up on persistence	75 (40–100)	90 (67–100)	0.04
N° (%) of patients with length on persistence <50%^b^	11 (36.7)	7 (11.7)	0.01

Data presented as median (Q_25_-Q_75_) unless otherwise indicated

^a^All the patients and controls achieved at least one DAS28 < 2.6; 2 cases abandoned health care immediately after entering the cohort; they were discarded from the analysis of flares along with their corresponding controls; date presented are from 33 cases and 66 controls

^b^At least 6 months of follow-up were required to evaluate persistence; 5 cases with insufficient follow-up and their corresponding controls were discarded. Data presented are obtained from 30 cases and 60 controls

The analysis was repeated considering cumulative data restricted to the year previous to HDO (or equivalent follow-up in controls). Nine cases with unavailable information due to early drop out (i.e., before the first year of follow-up was completed) and their corresponding controls were excluded. As described in Table 3, patients with HDO experienced more disease activity, more flares and they achieved sustained remission less frequently than their counterparts. They also had more scheduled visits, tended to receive more DMARDs and frequently tended to be less persistent with therapy.

**Table 3:** Cumulative disease activity, quality of life, treatment and persistence between cases and paired controls (Restricted*)

	Patients with HDO, *N* = 26	Patients with continuous follow-up, *N* = 52	*p*-value
Cumulative^a^ disease activity and function			
DAS28	2.8 (1.5–3.2)	1.7 (1.2–2.2)	0.004
N° of flares/patient	1 (1–2)	0 (0–1)	0.000
N° (%) of patients with sustained remission at outcome	17 (65.4)	48 (92.3)	0.007
SF36 score	86 (70.6–91.8)	89.3 (81.1–92)	0.12
N° of schedule visits	4 (3–4)	3 (3–4)	0.02
Cumulative^a^ treatment			
N° (%) of patients on corticosteroids	9 (34.6)	23 (44.2)	0.47
N° of DMARD/patient	1 (1–2)	1 (1–1)	0.10
Cumulative^a^ patient’s treatment behavior			
% of follow-up on persistence	82.9 (45–100)	84.5 (65–100)	0.38
N° (%) of patients with persistence <50%	7 (26.9)	6 (11.5)	0.11
N° (%) of patients recurrent non-persistent	8 (30.8)	19 (36.5)	0.8

Data presented as median (Q_25_-Q_75_) unless otherwise indicated

^a^Restricted to the year previous to HDO (or equivalent among the controls)

### Predictors of HDO (objective 3)

Table 4 summarizes adjusted and unadjusted analysis for baseline (age, comorbidities and years of education) and cumulative variables (related to disease activity, to treatment, to function, and to persistence) to be included in the Cox regression models, selected based on their clinical or statistical significance.

**Table 4:** Effect size for baseline and cumulative predictors of HDO

	Unadjusted analysis			Adjusted analysis		
	HR	95% CI	*p*	HR	95% CI	*p*
Baseline variables						
Age, years	1	1–1.03	0.99	1	1–1.1	0.83
Years of formal education	0.9	0.9–1	0.16	0.9	0.8–1	0.18
Presence of comorbidities	0.6	0.5–3.7	0.28	0.5	0.2–1.1	0.10
SF-36	1	0.96–1	0.08	1	1–1.02	0.79
Disease duration, months	0.9	0.8–10.5	0.18	0.9	0.8–1.1	0.46
Cumulative variables						
DAS28	1.3	1.1–1.7	0.003	1.2	0.9–1.6	0.40
N° of disease flares/patient^a^	2.9	1.2–5.2	0.001	2.5	1.4–4.5	0.003
Sustained remission	0.4	0.1–1	0.05	0.3	0.2–2.1	0.36
N° of DMARDs/patient	3.1	0.1–5.8	0.05	1.9	1.6–5.3	0.14
Persistence <50%^b^	2.7	1.3–5.7	0.006	1.7	0.7–4.3	0.24

*Data presented as HR, 95% CI, *p*-value

^a^All the patients and controls achieved at least one DAS28 < 2.6

^b^At least 6 months of follow-up were required to evaluate persistence; 5 cases with insufficient follow-up and their corresponding controls were discarded. Data presented are obtained from 30 cases and 60 controls

The different models tested included baseline and cumulative variables. The number of patients included in the models was 90 among whom 30 had HDO (at least 6 months of follow-up were required to evaluate persistence; 5 cases with insufficient follow-up and their corresponding controls were discarded). Cumulative variables restricted to the year previous to outcome had moderate to high correlations with cumulative variables up to HDO; accordingly, only those from one of the two categories were tested in the models. Results were consistent and are summarized in the Table 5. The cumulative number of flares (switched to achieving sustained remission or to DAS28-ESR), cumulative DMARD/patient and cumulative persistence < 50% emerged as predictors. Interestingly, in the 3 models, persistence < 50% had the greatest impact followed by number of DMARDs/patient and by the level of disease activity evaluated. Similar results were obtained when the cumulative period was limited to the previous year’s outcome (data not shown).

**Table 5:** Predictors of HDO

	Model 1^a^			Model 2^a^			Model 3^a^		
	OR	95% CI	*p*	OR	95% CI	*p*	OR	95% CI	*p*
N° of disease flares	2.5	1.4–4.5	0.003						
Sustained remission				0.4	0.1, 0.9	0.031			
DAS28							2.1	1.3–3.3	0.002
N° of DMARDs/patient	2.9	1.6–5.3	0.001	1.7	1.0–3.1	0.06	1.7	1–3.0	0.05
Persistence < 50%	3.01	1.3–7.3	0.011	2.2	1.0–5.2	0.06	1.9	1–4.6	0.06

Model 1: Comorbidities (or age), SF36, disease flares, number of DMARDs/patient and persistence <50%

Model 2: Age (or comorbidities), remission, number of DMARDs/patient and persistence <50% and number of visits

Model 3: Age (or comorbidities), DAS28-ESR, number of DMARDs/patient and persistence <50% and number of visits

^a^Data presented as HR, 95% CI, *p*-value. Ninety patients were included, 30 of them with HDO and 60 with complete follow-up

### Impact of HDO on patient’s outcomes (objective 4)

Five HDO patients returned to the clinic: 4 (80%) of whom were female, and the group had a mean ± SD age of 30.9 ± 6.7 years and a median drop out time of 3.8 years (2.3 to 5.8 years).

**Fig. 1:**
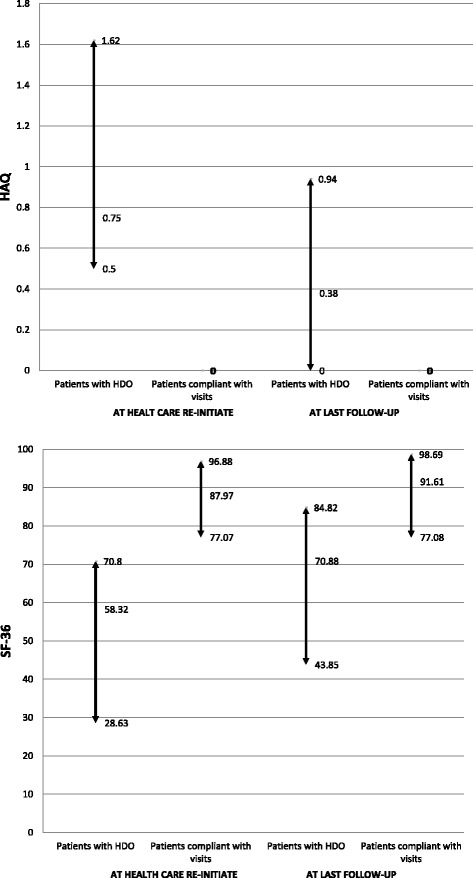
Impact of HDO on health related quality of life outcomes. Comparison of HAQ (*upper panel*) and SF-36 (*bottom panel*) scores (median, [Q_25_-Q_75_]) between cases (patients with HDO) and controls (patients compliant with schedule visits) at health care re-initiate and at last follow-up

HAQ and SF-36 scores were compared, as were erosions between the five HDO patients who returned to the clinic and their respective paired controls. As shown in Fig. 1, patients from the former group had higher HAQ and poorer SF-36 than their counterparts, either when they returned to the clinic (or equivalent time in those with continuous follow-up) or at last follow-up evaluation. Also, patients from the former group frequently had more erosions at return to the clinic (40% versus 26%; *p* = 0.61) and at last follow-up (60% versus 26.7%; *p* = 0.29), although the differences were not statistically significant.

## Discussion

Early intervention in RA according to a “Treat to target” strategy improves disease outcomes [23, 24] and initial assessment by a rheumatologist as early as possible is believed to be essential [25], in addition to appropriate follow-up. Nevertheless, after initial care for several months, a sizeable proportion of patients decide not to return to the clinic, with potentially disastrous consequences to their health-related quality of life [26].

The present study involved a well-characterized cohort of Mexican Mestizo patients with early disease and standardized follow-ups up to 10 years. We found that 20.6% of the patients dropped out of health care, which closely approximates the proportion published by Nell-Duxneuner et al. [26], who reported on a population of 119 patients with inflammatory arthritis of ≤12 weeks’ duration, among whom, 67 had an RA diagnosis. Welsing et al. [27] initiated an inception cohort of early RA in 1985 and subsequently reported five years dropout rates between 21.3% and 25.4% in three subcohorts defined based on the date of patient inclusion. Among the predominant reasons for dropout were death (20% to 25%), voluntary withdrawal (20% to 30%) and voluntary withdrawal due to low disease activity (5% to 10%) [28]. Van Aken et al. [29] initiated an EAC in 1993, with follow-up visits planned after three and six months, and every year thereafter, and their three- to four-year dropout rate was 25%. A randomized clinical trial with long follow-up duration provided data regarding continued participation, with a completeness percentage of 60% after 10 years [30]. Nonetheless, among the reasons for drop-out were ‘revised diagnosis’ and ‘deaths’, neither of which was included in our study. In two other intervention trials performed in early RA [31, 32], 71% and 61% of patients, respectively, responded to recall after 11 years. It is worth mentioning that randomized clinical trials have differential characteristics that impact patients’ rate of abandon.

We also found that the vast majority of patients who dropped out of health care did so during the first five years of follow-up, similar to Reisine et al. [33] who reported that only 46% of RA patients remained in their study, and a greater drop-out rate was observed over the first several years compared with the last several years.

Factors contributory to HDO were related to insufficient control of disease activity, complexity of treatment regimens and to a suboptimal length of time of persistence with such regimens. The present study was the first to include cumulative variables and consider their behaviour throughout the entire patient follow-up. This distinctive characteristic of our study may explain differences with the limited number of published studies that have addressed the topic. Markusse et al. [30] reported that older patients and worse functional ability predicted drop out, while reporting more adverse events and exhibiting more absolute radiographic damage and damage progression correlated with study continuation. Also, in addition to psychosocial and socioeconomic factors, Reisine et al. [33] reported that having two to three joint groups with flares (versus four to five joint groups) was associated with continued study participation. In contrast, in a real-life setting of a very early arthritis clinic, Nell-Duxneuner et al. [26] found that measures of disease activity were better in attending patients than non-attending patients. It may be argued that dissimilar results across studies may be explained by differences in RA duration, in how disease activity was assessed and treatment recorded, or in the different follow-up for time dependent variables. We also believe that, contrary to the ‘real world’, clinical trials are developed in uniform but particularly social, economic and clinical contexts that impact continued patient participation [34].

Finally, we confirmed that inappropriate termination with health service contact affected patient outcomes. Although this finding may be intuitive, a higher percentage of drug-free remission had been described in patients attending EAC, reflecting that patients with more benign forms of arthritis or self-limiting disease are regularly included and followed (until unnecessary) in such clinics [26]. Patients enrolled in our EAC had a high percentage of disease-specific autoantibodies that had been associated to unfavorable outcomes [35, 36]. Such patients benefit most from therapeutic intervention and close disease monitoring by a rheumatologist [37, 38].

Limitations of the present study should be addressed. We could not ensure that patients included in the drop-out group had discontinued all ongoing contact with health services (unless for the limited number of patients who returned to the clinic). We investigated a limited number of potential baseline and cumulative predictors of HDO. Psychosocial factors are important determinants in treatment discontinuation and HDO [39, 40] and our EAC does not perform formal psychiatric evaluations. Termination of health care may be assumed to represent a behavioural sign of dissatisfaction and this outcome was not conveniently evaluated. The number of HDO patients who returned to the clinic was small and results should be interpreted with caution. Finally, patients from our inception cohort had particular sociodemographic characteristics, ethnicity and treatment, and the EAC belongs to a particular health system and results may not be generalized to populations with dissimilar characteristics.

## Conclusions

In conclusion, 20.6% of RA patients attending a real-life EAC setting dropped out of health care over a 10-year follow-up period. Disease activity, intensive treatment and insufficient compliance with therapy accumulated during patient follow-up predicted HDO. Abandonment of health care had a negative impact on patient outcomes and was sustained even after health care was reinitiated.

## Abbreviations

ACCP: antibodies to citrullinated peptides
ACR: American College of Rheumatology
CQ: Concordance Questionnaire
CRP: C-reactive protein
DAS28-ESR: Disease Activity Score, 28 joints evaluated with ESR
DMARD: disease-modifying anti-rheumatic drug
EAC: early arthritis clinic
ESR: erythrocyte sedimentation rate
HAQ: health assessment questionnaire
HDO: Health care drop out
Non-P: Non-persistent
RA: rheumatoid arthritis
RF: rheumatoid factor
SD: Standard Deviation
SF-36: Short-form 36 Health Survey

## References

[1] Kosinski M, Kujawski SC, Martin R, Wanke LA, Buatti MC, Ware JE (2002). Health-related quality of life in early rheumatoid arthritis: impact of disease and treatment response. Am J Manag Care.

[2] Sanderson T, Kirwan J (2009). Patient-reported outcomes of arthritis: time to focus on personal life impact measures?. Editorial Arthritis Rheum.

[3] Wolfe F, Mitchell DM, Sibley JT, Fries JF, Bloch DA, Williams CA (1994). The mortality of rheumatoid arthritis. Arthritis Rheum.

[4] Mody GM, Cardiel MH (2008). Challenges in the management of rheumatoid arthritis in developing countries. Best Pract Res Clin Rheumatol.

[5] Pascual-Ramos V, Contreras-Yáñez I, Villa AR, Cabiedes J, Rull-Gabayet M (2009). Medication persistence over two years of follow-up in a cohort of early rheumatoid arthritis patients: associated factors and relationship with disease activity and disability. Arthritis Res Ther.

[6] de Klerk E, van der Heijde D, Landewé R, van der Tempel H, Urquhart J, van der Linden S (2003). Patient compliance in rheumatoid arthritis, polymyalgia Rheumatica, and gout. J Rheumatol.

[7] Gossec L, Tubach F, Dougados M, Ravaud P (2007). Reporting of adherence to medication in recent randomized controlled trials of 6 chronic diseases: a systematic literature review. Am J Med Sci.

[8] Deyo RA, Inui TS, Sullivan B (1981). Noncompliance with arthritis drugs: magnitude, correlates, and clinical implications. J Rheumatol.

[9] Tuncay R, Eksioglu E, Cakir B, Gurcay E, Cakci A (2007). Factors affecting drug treatment compliance in patients with rheumatoid arthritis. Rheumatol Int.

[10] García-González A, Richardson M, García Popa-Lisseanu M, Cox V, Kallen MA, Janssen N (2008). Treatment adherence in patients with rheumatoid arthritis and systemic lupus erythematosus. Clin Rheumatol.

[11] Lorish CD, Richards B, Brown S (1989). Missed medication doses in rheumatic arthritis patients: intentional and unintentional reasons. Arthritis Care Res.

[12] Lorish CD, Richards B, Brown S (1990). Perspective of the patient with rheumatoid arthritis on issues related to missed medication. Arthritis Care Res.

[13] Viller F, Guillemin F, Briançon S, Moum T, Suurmeijer T, van den Heuvel W (1999). Compliance to drug treatment of patients with rheumatoid arthritis: a 3 year longitudinal study. J Rheumatol.

[14] Viller F, Guillemin F, Briançon S, Moum T, Suurmeijer T, van den Heuvel W (2000). Compliance with drug therapy in rheumatoid arthritis. A longitudinal European study. Joint Bone Spine.

[15] Grijalva CG, Chung CP, Arbogast PG, Stein CM, Mitchel EF, Griffin MR (2007). Assessment of adherence to and persistence on disease-modifying anti-rheumatic drugs (DMARDs) in patients with rheumatoid arthritis. Med Care.

[16] Curkendall S, Patel V, Gleeson M, Campbell RS, Zagari M, Dubois R (2008). Compliance with biologic therapies for rheumatoid arthritis: do patients out-of-pocket payments matter?. Arthritis Care Res.

[17] Contreras-Yáñez I, Ponce de León S, Cabiedes J, Rull-Gabayet M, Pascual-Ramos V (2010). Inadequate therapy behavior is associated to disease flares in patients with rheumatoid arthritis who have achieved remission with disease modifying antirheumatic drugs. Am J Med Sc.

[18] Contreras-Yáñez I, Cabiedes J, Villa AR, Rull-Gabayet M, Pascual-Ramos V (2010). Persistence on therapy is a major determinant of patient-, physician- and laboratory- reported outcomes in recent-onset rheumatoid arthritis patients. Clin Exp Rheumatol.

[19] Pascual-Ramos V, Contreras-Yáñez I (2013). Motivations for inadequate persistence with disease modifying antirheumatic drugs. The patient’s perspective. BMC Musculoskelet Disord.

[20] Lim TO, Ngah BA (1991). The Mentakab hypertension study project. Part II- why do hypertensives drop out of treatment?. Singap Med J.

[21] Prevoo ML, van’t Hof MA, Kuper HH, van Leeuwen MA, van de Putte LB, van riel PL (1995). modified disease activity score that include twenty-eight-joint counts. Development and validation in a prospective longitudinal study of patients with rheumatoid arthritis. Arthritis Rheum.

[22] Contreras-Yáñez I, Pascual-Ramos V (2015). Window of opportunity to achieve major outcomes in early rheumatoid arthritis patients: how persistence with therapy matters. Arthritis Res Ther.

[23] van Gestel AM, Prevoo ML, van’t Hof MA, van Rijswijk MH, van de Putte LB, van riel PL (1996). development and validation of the European league against rheumatism response criteria for rheumatoid arthritis. Comparison with the preliminary American College of Rheumatology and the World Health Organization/international league against rheumatism criteria. Arthritis Rheum.

[24] Emery P, Breedveld FC, Dougados M, Kalden JR, Schiff MH, Smolen JS (2002). Early referral recommendation for newly diagnosed rheumatoid arthritis: evidence based development of a clinical guide. Ann Rheum Dis.

[25] Combe B, Landewe R, Lukas C, Bolosiu HD, Breedveld F, Dougados M (2007). EULAR recommendations for the management of early arthritis: report of a task force of the European standing Committee for International Clinical Studies Including Therapeutics (ESCISIT). Ann Rheum Dis.

[26] Nell-Duxneuner V, Rezende LS, Stamm TA, Duer M, Smolen JS, Machold KP (2012). Attending and non-attending patients in a real-life setting on an early arthritis clinic: why do people leave clinics and where do they go?. Clin Exp Rheumatol.

[27] Welsing PM, van Riel PL (2004). The Nijmegen inception cohort of early rheumatoid arthritis. J Rheumatol Suppl.

[28] Welsing PM, Fransen J, van Riel PL (2005). Is the disease course of rheumatoid arthritis becoming milder? Time trends since 1985 in an inception cohort of early rheumatoid arthritis. Arthritis Rheum.

[29] van Aken J, Lard LR, le Cessie S, Hazes JM, Breedveld FC, Huizinga TW (2004). Radiological outcome after four years of early versus delayed treatment strategy in patients with recent onset rheumatoid arthritis. Ann Rheum Dis.

[30] Markusse IM, Dirven L, Han KH, Ronday HK, Kerstens PJ, Lems WF (2015). Continued participation in a ten-year tight control treat-to-target study in rheumatoid arthritis: why keep patients doing their best?. Arthritis Car Res.

[31] Rantalaiho V, Korpela M, Hannonen P, Kautiainen H, Jarvenpaa S, Leirisalo-Repo M (2009). The good initial response to therapy with the combination of traditional disease modifying anti-rheumatic drugs is sustained over time: the eleven-year results of the Finnish Rheumatoid Arthritis Combination Therapy Trial. Arthritis Rheum.

[32] van Tuyl LH, Boers M, Lems WF, Landewé RB, Han H, van der Linden S (2010). Survival, comorbidities, and joint damage 11 years after the COBRA combination therapy trial in early rheumatoid arthritis. Ann Rheum Dis.

[33] Reisine S, Fifield J, Winkelman DK (2000). Characteristics of rheumatoid arthritis patients: who participates in long-term research and who drops out?. Arthritis Car Res.

[34] Udrea G, Dumitrescu B, Purcarea M, Balan I, Rezus E, Deculescu D (2009). Patient’s perspectives and motivators to participate in clinical trials with novel therapies for rheumatoid arthritis. J Med Life.

[35] van der Helm-van Mil AH, le Cessie S, van Dongen H, Breedveld FC, Toes RE, Huizinga TW (2007). A prediction rule for disease outcome in patientes with recent-onset undifferentiatied arthritis: how to guide individual treatment decisions. Arthritis Rheum.

[36] de Vries-Bouwstra J, Le Cessie S, Allaart C, Breedveld F, Huizinga T (2006). Using predicted disease outcome to provide differentiated treatment of early rheumatoid arthritis. J Rheumatol.

[37] Harrison BJ, Symmons DP, Brenan P, Barret EM, Silman AJ (1996). Natural remission in inflammatory polyarthritis: issues of definition and prediction. Br J Rheumatol.

[38] Strangfeld A, Zink A (2005). Comparison of treatment, and outcome of rheumatological and non-rheumatological care in patients with rheumatoid arthritis. Rheumatology.

[39] Wong M, Mulherin D. The influence of medication beliefs and other psychosocial factors on early discontinuation of disease-modifying anti-rheumatic drugs. Musculoskeletal Car. 2007;(5):148–59. 10.1002/msc.107 17590885

[40] Rossi A, Amaddeo F, Bisoffi G, Ruggeri M, Thornicroft G, Tansella M (2002). Dropping out of care: inappropriate terminations of contact with community-based psychiatric services. Br J Psych.

